# *Streptacidiphilus hamsterleyensis* sp. nov., isolated from a spruce forest soil

**DOI:** 10.1007/s10482-013-0015-1

**Published:** 2013-08-30

**Authors:** Patrycja Golinska, Byung-Yong Kim, Hanna Dahm, Michael Goodfellow

**Affiliations:** 1 School of Biology, University of Newcastle, Newcastle upon Tyne, NE1 7RU UK; 2 Department of Agricultural Microbiology, National Academy of Agricultural Science, Rural Development Administration, Suwon, 441-707 South Korea; 3 Department of Microbiology, Nicolaus Copernicus University, 87-100 Toruń, Poland

**Keywords:** Actinobacteria, Polyphasic taxonomy, *Streptacidiphilus hamsterleyensis* sp. nov., Spruce litter

## Abstract

Three acidophilic actinobacteria, isolates LSCA2, FGG8 and HSCA14^T^, recovered from spruce litter were examined using a polyphasic approach. Chemotaxonomic and morphological properties of the isolates were found to be consistent with their classification in the genus *Streptacidiphilus*. The isolates were shown to have identical 16S rRNA gene sequences and were most closely related to *Streptacidiphilus neutrinimicus* DSM 41755^T^ (99.9 % similarity). However, DNA:DNA relatedness between isolate HSCA14^T^ and the type strain of *S. neutrinimicus* was found to be low at 44.0 (±14.1) %. A combination of phenotypic features, including degradative and nutritional characteristics were shown to distinguish the isolates from their nearest phylogenetic neighbours. Data from this study show that the isolates form a novel species in the genus for which the name *S. hamsterleyensis* sp. nov. is proposed. The type strain is HSCA 14^T^ (=DSM 45900^T^ = KACC 17456^T^ = NCIMB 14865^T^).

**Electronic supplementary material:**

The online version of this article (doi:10.1007/s10482-013-0015-1) contains supplementary material, which is available to authorized users.

## Introduction

The genus *Streptacidiphilus*, a member of the family *Streptomycetaceae*, was proposed by Kim et al. (2003) for actinobacteria that grow between pH 3.5 and 6.0; form aerial hyphae that differentiate into long chains of flexuous, smooth-surfaced spores; contain major proportions of LL-diaminopimelic acid, galactose and mannose in whole-organism hydrolysates; saturated, *iso*- and *anteiso*-fatty acids; hexa- and octahydrogenated menaquinones with nine isoprene units as predominant isoprenologues; and complex polar lipid patterns which include diphosphatidylglycerol, phosphatidylethanolamine, phosphatidylinositol and phosphatidylinositol mannosides. Streptacidiphili are common in acidic soils and coniferous litter (Golinska et al. 2013a).

It is important to clarify the taxonomy of acidophilic sporoactinobacteria as they are a source of antifungal agents (Williams and Khan 1974), have a role in the turnover of organic matter at low pH values (Goodfellow and Williams 1983; Williams et al. 1984) and produce chitinases and diastases with pH optima below those of neutrotolerant streptomycetes (Williams and Flowers 1978; Williams and Robinson 1981). The genus currently contains nine validly named species (Cho et al. 2008; Golinska et al. 2013a) although there is evidence that it is underspeciated (Lonsdale 1985; Goodfellow and Simpson 1987; Seong et al. 1993, 1995). *Streptacidiphilus* species are closely related on the basis of 16S rRNA gene sequence data but are also very similar to the members of the genera *Kitasatospora* and *Streptomyces* (Kämpfer 2012a; Labeda et al. 2012), the two other members of the family *Streptomycetaceae*. The case for the recognition of these taxa as sister genera is supported by sequence data of conserved proteins which show that *Kitasatospora* is significantly different from *Streptomyces* (Girard et al. 2013).

The present study is a continuation of our bioprospecting studies on acidophilic and aciditolerant actinobacteria isolated from a spruce forest soil. Several isolates were considered to have colonial properties typical of streptacidiphili, three of which were the subject of a polyphasic taxonomic study. The resultant data showed that the isolates belong to a new *Streptacidiphilus* species, *Streptacidiphilus hamsterleyensis* sp. nov.

## Materials and methods

### Organisms, maintenance and biomass preparation

The three organisms, strains LSCA2, FGG8 and HSCA14^T^, were isolated from the litter, fermentation and humus layers respectively of a spruce soil at Hamsterley Forest; the site and the dilution plate procedures have been described previously (Golinska et al. 2013a, b). The strains were isolated from starch-casein plates (Kűster and Williams 1964) using either agar (SCA) or gellan gum (GG) as gelling agents. They were maintained on acidified modified Bennett’s agar (Jones 1949) at room temperature and as hyphal fragments and spores in glycerol (v/v) at −80 °C.

Biomass for the chemotaxonomic and molecular systematic studies was prepared by growing the isolates in shake flasks of acidified glucose-yeast extract broth (pH 5.5; Gordon and Mihm 1962) at 150 revolutions per minute for 3 weeks at 28 °C. Cells were harvested by centrifugation and washed twice in distilled water; biomass for the chemotaxonomic analyses was freeze-dried and that for the molecular work stored at −20 °C. Biomass for the fatty acid analysis carried out on isolate HSCA14^T^ was harvested from modified Bennett’s broth (Jones 1949), adjusted to pH 5.5, following incubation at 28 °C for 7 days.

### Phylogenetic analyses

Extraction of genomic DNA, PCR-mediated amplification of the 16S rRNA genes of the three isolates and direct sequencing of the purified PCR products were carried out as described previously (Golinska et al. 2013a, b). The closest phylogenetic neighbours based on 16S rRNA gene similarities were sought using the EzTaxon server (http://eztaxon-e.ezbiocloud.net/; Kim et al. 2012). The resultant 16S rRNA gene sequences were aligned with sequences of all validly named species of the genus *Streptacidiphilus* using ClustalW. Phylogenetic analyses were carried out using MEGA5 (Tamura et al. 2011) and PHYML (Guindon and Gascuel 2003) software packages. Evolutionary distances were generated for the neighbour-joining, maximum-likelihood and maximum-parsimony methods as described by Jukes and Cantor (1969). The tree topologies were evaluated by a bootstrap analysis (Felsenstein 1985) of the neighbour-joining data based on 1,000 resamplings using MEGA5 software. The root position of unrooted trees were estimated using the sequence of *Streptomyces albus* subsp. *albus* DSM 40313^T^ (GenBank accession number AJ 621602).

### DNA:DNA relatedness

The DNA:DNA relatedness value (∆Tm) between isolate HSCA14^T^ and *Streptacidiphilus* *neutrinimicus* DSM 41755^T^ was determined, in duplicate, using a fluorimetric method (Gonzalez and Saiz-Jimenez 2005). The optimal temperature for renaturation (Tm) was calculated using the equation *Tor* − 0.51 (% GC) + 47. The melting temperatures (Tm) at which 50 % of the initial double stranded DNA denatured into single-stranded DNA for isolate HSCA14^T^ and hybrid DNA of the isolate HSCA14^T^: *S. neutrinimicus* DSM 41755^T^ were compared and the differences (∆Tm) calculated.

### Chemotaxonomy

The three isolates were examined for chemical properties known to be of value in the systematics of genera classified in the family *Streptomycetaceae* (Kämpfer 2012a, b). Standard chromatographic procedures were used to determine the isomers of diaminopimelic acid (Staneck and Roberts 1974), isoprenoid quinones (Collins 1985), polar lipids (Minnikin et al. 1984) and whole-organism sugars (Hasegawa et al. 1983), using appropriate controls. Cellular fatty acids of isolate HSCA14^T^ were extracted, methylated and determined by gas chromatography (Hewlett Packard instrument 6890) and analysed using the standard Sherlock Microbial Identification (MIDI) system, version 5 (Sasser 1990). The G+C mol% of the DNA of strain HSCA14^T^ was determined following the procedure described by Gonzalez and Saiz-Jimenez (2002).

### Cultural and morphological properties

The isolates were examined for cultural and morphological properties following growth on acidified International Streptomyces Project (ISP) media (Shirling and Gottlieb 1966), as described previously (Golinska et al. 2013a). Hyphal and spore chain arrangements were detected on acidified oatmeal agar (ISP medium 3; Shirling and Gottlieb 1966) following incubation at 28 °C for 14 days, using the cover slip method of Kawato and Shinobu (1959). The arrangement and surface ornamentation of isolate HSCA14^T^ were detected by examining a gold-coated dehydrated preparation from the acidified oatmeal agar plate with a scanning electron microscope (Cambridge Stereoscan 240) and the procedure described by O’Donnell et al. (1993).

### Phenotypic tests

A broad range of phenotypic tests were carried out on the isolates using media and methods described by Williams et al. (1983) but with acidified media. The isolates were also examined for their ability to grow at various temperatures (10, 30, 35 and 40 °C), pH values (4, 5, 6 and 7) and sodium chloride concentrations (1, 3, 5, 7 and 10 %, w/v) using acidified modified Bennett’s agar (Jones 1949).

## Results and discussion

Surprisingly little is known about acidophilic filamentous actinobacteria even though they were discovered a long time ago (Jensen 1928), are common in acidic habitats (Williams et al. 1971; Khan and Williams 1975; Goodfellow and Dawson 1978; Goodfellow and Simpson 1987) and may well be a source of acid stable antibiotics and enzymes (Williams and Khan 1974; Williams and Flowers 1978). The results of the present study provide further evidence that the acidiphilic taxon *Streptacidiphilus* is underspeciated and common in coniferous litter (Lonsdale 1985; Golinska et al. 2013a).

### Chemotaxonomic, cultural and morphological properties

The three strains isolated from spruce litter taken from Hamsterley Forest were found to have genotypic and phenotypic properties consistent with their classification in the genus *Streptacidiphilus* (Kim et al. 2003; Golinska et al. 2013a). They were shown to be aerobic, Gram-positive, non-acid- alcohol-fast actinobacteria which form extensively branched substrate mycelia that carried abundant white to gray aerial spore mass on oatmeal agar. The strains were found to grow well on most of the ISP media tending to form a gray aerial spore mass and yellowish substrate mycelia (Table 1). The isolates LSCA2, FGG8 and HSCA14^T^ were also shown to have whole-organism hydrolysates rich in LL-diaminopimelic acid, galactose and rhamnose, major proportions of hexa- and octahydrogenated menaquinones with nine isoprene units (in ratios of 1:1.2; 1:1.4 and 1:1.8, respectively), and diphosphatidylglycerol, phosphatidylethanolamine (diagnostic marker), phosphatidylinositol and phosphatidylinositol mannosides as predominant polar lipid components (phospholipid pattern 2 sensu Lechevalier et al. 1977; Online supplementary Fig. 1). The fatty acid profile of isolate HSCA14^T^ was shown to contain major proportions (>10 %) of *iso*-C_15:0_ (14.1 %), *anteiso*-C_15:0_ (21.7 %), *iso*-C_16:0_ (19.3 %) and C_16:0_ (16.9 %), minor proportions (>1.5 %) of *iso*-C_14:0_ (3.9 %), C_14:0_ (1.5 %), *iso*-C_17:0_ (3.5 %), *anteiso*-C_17:0_ (8.2 %), C_17: *cyclo*_ (5.6 %), summed features C_16:1_ ω7c/C_16:1_ ω6c (1.3 %) and trace amounts (<0.8 %) of other components (fatty acid type 2c, Kroppenstedt 1985). Isolate HSCA14^T^ was determined to have a DNA G+C base composition of 71.0 mol%.

**Table 1:** Growth and cultural characteristics of isolates on acidified ISP media after incubation for 3 weeks at 28 °C

Medium	Isolates		
	LSCA2, FGG8, HSCA14^T^		
	Growth	Colour of aerial mass	Colour of substrate mycelium
Tryptone-yeast extract agar (ISP 1)	+	Light gray	Grayish yellow
Yeast extract-malt extract agar (ISP 2)	+++	Medium gray	Yellowish brown
Oatmeal agar (ISP 3)	++	White to gray	Dark orange yellow
Glucose-asparagine agar (ISP 5)	+++	Light gray	Light yellowish brown
Tyrosine agar (ISP 7)	++	Light gray	Light yellowish brown

The isolates did not produce diffusible pigments or grow on inorganic salts-starch agar (ISP4) or peptone-yeast extract agar (ISP 6)

+++ abundant, ++ moderate, + poor growth

### Phylogenetic analyses

**Fig. 1:**
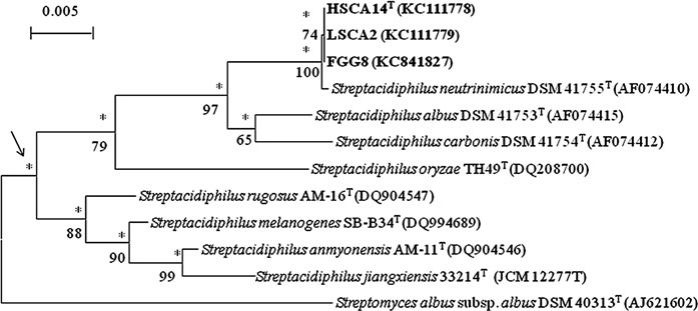
Neighbour-joining tree based on nearly complete 16S rRNA gene sequences (1,383–1,523 nucleotides) showing relationships between the isolates and between them and the type strains of *Streptacidiphilus* species*. Asterisks* indicate branches that were also found using the maximum-likelihood and maximum-parsimony tree-making algorithms. Numbers at the nodes indicate the percentage bootstrap values based on 1,000 re-sampled datasets, only values above 50 % are given. T, type strain. *Bar* 0.005 substitutions per nucleotide position. The root position of the tree was determined using *Streptomyces albus* subsp*. albus* DSM 40313^T^ as outgroup

Almost complete 16S rRNA gene sequences of the isolates (1403–1408 nucleotides [nt]) were generated; the isolates were shown to have identical 16S rRNA gene sequences (Genbank Accession numbers KC111778, KC111779 and KC841827) and to form a branch in the *Streptacidiphilus* gene tree that was supported by all of the tree-making algorithms and by a 100 % bootstrap value (Fig. 1). The isolates were also shown to form a subclade in the *Streptacidiphilus* 16S rRNA gene tree together with the type strains of *S. albus* (type species), *S. carbonis* and *S.* *neutrinimicus*; the taxonomic integrity of this subclade was supported by a 97 % bootstrap value and by all of the tree-making algorithms (Fig. 1). In turn, the isolates were found to be most closely related to *S. neutrinimicus* DSM 41755^T^, these organisms were shown to share a 16S rRNA gene similarity of 99.9 %, a value equivalent to a single nucleotide difference. Corresponding 16S rRNA gene sequence similarities with the type strains of *S. albus* and *S. carbonis* were 98.4 and 98.6 %, values shown to correspond to 23 and 20 nt differences, respectively. The similarities of 16S rRNA gene sequences between the isolate and the type strains of the remaining *Streptacidiphilus* species were found to range from 94.4 to 97.0 %.

The ΔTm value between isolate HSCA14^T^ g DNA and isolate HSCA14^T^/*S. neutrinimicus* and DSM 41755^T^ hybrid DNA was found to be 9 (±2.8) °C, a result that corresponds to a DNA:DNA similarity of 44 (±14.1) % according to Gonzalez and Saiz-Jimenez (2005) i.e. well below the 70 % cut-off point recommended for assigning bacterial strains to the same genomic species (Wayne et al. 1987). The phenomenon of very high 16S rRNA gene sequence similarities between species distinguishable by DNA:DNA similarity is not uncommon in the *Streptomycetaceae* particularly amongst tight 16S rRNA gene clades, for example such as the *Streptomyces violaceusniger* clade (Goodfellow et al. 2007).

### Phenotypic tests

The phenotypic properties of the isolates were compared with those of the type strains of *S. albus*, *S. carbonis* and *S. neutrinimicus* which had been studied previously using the same media and methods (Kim et al. 2003; Cho et al. 2008). It can be seen from Table 2 that the isolates can be distinguished from the type strains of their closest phylogenetic neighbours using a broad range of properties. Thus, the isolates, unlike *S. neutrinimicus* DSM 41755^T^, are able to metabolise Tweens 40 and 60, use d-melezitose, α-methyl-d-glucoside and l-rhamnose as sole carbon sources and grow at 10 and 30 °C. In turn, the *S. neutrinimicus* type strain, unlike the isolates, degrades starch, grows on sodium pyruvate and sodium succinate as sole carbon sources and at pH 4.0. All of the strains are able to use l-arabinose, glycerol, glycogen, d-melibiose and d-raffinose as sole carbon sources, but not amygdalin, sodium adipate or sodium oxalate.

**Table 2:** Phenotypic properties that distinguish the isolates from the type strains of their nearest phylogenetic neighbours

Characteristics	Isolates LSCA2, FGG8, HSCA14^T^	*S. albus* DSM 41753^T^	*S. carbonis* DSM 41744^T^	*S. neutrinimicus* DSM 41755^T^
Growth on acidified oatmeal agar				
Aerial spore mass	White	White	White	White/greyish white
Substrate mycelium	Light yellowish brown	Cream	Cream	Cream
Degradation of				
Starch	−	+	+	+
Tween 40	+	−	−	−
Tween 60	+	+	+	−
Xanthine	−	+	+	−
Growth on sole carbon Sources				
At 1 %, w/v				
d-glucosamine	+	+	−	+
*Myo*-inositol	−	−	+	−
Inulin	+	−	−	+
d-melezitose	+	−	−	−
alpha-methyl-d-glucoside	+	−	−	−
l-rhamnose	+	+	+	−
d-xylose	+	−	+	+
At 0.1 %, w/v				
Sodium pyruvate	−	+	+	+
Sodium succinate	−	+	+	+
Growth on l-isoleucine as a sole nitrogen source				
(0.1 %, w/v)	−	+	+	−
Growth at				
pH 4.0	−	+	+	+
pH 6.0	+	+	+	−
10 °C	+	−	−	−
30 °C	+	−	+	−
G+C contents of DNA (mol %)	71	70–72	70–72	70–72
Predominant phospholipids	DPG, PE, PI, PIM’S	DPG, PE, PI, PIM’S	DPG, PE, PI, PIM’S	DPG, PE, PI, PIM’S

Data for the type strains of *S. albus*, *S. carbonis* and *S. neutrinimicus* were taken from Kim et al. (2003) and Cho et al. (2008)

+ positive, − negative

## Conclusions

The chemotaxonomic, phenotypic and phylogenetic characteristics of isolates LSCA2, FGG8 and HSCA14^T^ show that they represent a novel species for which the name *S. hamsterleyensis* is proposed.

### Description of *Streptacidiphilus hamsterleyensis* sp. nov.

*Streptacidiphilus hamsterleyensis* (ham.ster.ley.en’sis. N.L. masc. adj. *hamsterleyensis*, belonging to Hamsterley Forest in County Durham in the North East of England, the source of the isolate).

Aerobic, Gram-positive, non-acid- alcohol-fast, acidophilic actinobacteria which form an extensively branched substrate mycelium that carries aerial hyphae that differentiate into long straight to flexuous chains of smooth, cylindrical spores (0.6 × 0.8 μm; Online supplementary Fig. 2). Grows at 10–30 °C, optimally ~25 °C, from pH 4.5 to 6.0, optimally ~pH 5.5 and in the presence of 1 % but not 3 % and higher sodium chloride (w/v). Gelatin and Tweens 40 and 60 are metabolized, but not casein, chitin, elastin, guanine, hypoxanthine, tyrosine, uric acid or xylan. Nitrate is reduced, but strains are negative for aesculin, allantoin, arbutin and urea, hydrolysis. d-cellobiose, d-fructose, d-galactose, d-glucosamine, d-glucose, d-lactose, d-maltose, d-raffinose, d-sucrose and d-trehalose are used as sole carbon sources for energy and growth, but not d- or l-arabitol, dextran, *meso*-erythritol, d-glucuronic acid, d-mannitol, d-salicin or xylitol (all at 1 %, w/v) or ethanol (1 %, v/v). Does not use acetate, benzoate, butyrate, citrate, fumarate, hippurate, or propionate (sodium salts) or *p*-hydroxybenzoic acid (all at 0.1 %, w/v) as sole sources of carbon. l-alanine is used as a sole nitrogen source, but not l-arginine, l-aspartic acid, l-cysteine, l-histidine, l-phenylalanine, l-threonine or l-valine (all at 0.1 %, w/v). l-asparagine, d-hydroxyproline and l-serine are metabolized as sole carbon and nitrogen sources, but not acetamide, l-aspartic acid, l-cysteine, l-histidine, l-isoleucine, l-methionine, l-phenylalanine, l-threonine or l-valine (all at 0.1 %, w/v) or ethanolamine (0.1 %, v/v). Additional phenotypic properties are given in the text and in Tables 1 and 2. The major fatty acids are *iso*-C_15:0_, *anteiso*-C_15:0_, C_16:0_ and *iso*-C_16:0_. Other chemotaxonomic properties are typical of the genus *Streptacidiphilus*. The G+C content of the DNA of the type strain is 71.0 mol%.

The species contains the type strain HSCA14^T^ (=DSM 45900^T^ = KACC 17456^T^ = NCIMB 14865^T^) and isolates FGG8 and LSCA2 which were isolated from the humus, fermentation and litter horizons of a spruce stand at Hamsterley Forest, County Durham, England. The Genbank Accession number of the 16S rRNA gene sequence of strain HSCA14^T^ is KC111778.

## Electronic supplementary material

Below is the link to the electronic supplementary material.

Fig. 1. Two dimensional thin-layer chromatography of polar lipids of isolate HSCA14^T^ stained with molybdenum blue (Sigma). Chloroform : methanol : water (32.5 : 12.5 : 2.0 v/v) were used in the first direction and chloroform : acetic acid : methanol : water (40 : 7.5 : 6 : 2 v/v) in the second direction. DPG, diphosphatidylglycerol; PE, phosphatidylethanolamine; PI, phosphatidylinositol; PIMS, phosphatidylinositol mannosides. Supplementary material 1 (PDF 133 kb)

Fig. 2. Scanning electron micrograph of isolate HSCA14^T^ showing straight chains of smooth-surfaced, cylindrical spores on oatmeal agar after growth for 3 weeks at 28 °C. Bar, 1.0 μm. Supplementary material 2 (PDF 115 kb)
