# Supplementary figures and images for: Streptacidiphilus hamsterleyensis sp. nov., isolated from a spruce forest soil
Source: Antonie Van Leeuwenhoek. 2013 Aug 30;104(6):965–72. doi: 10.1007/s10482-013-0015-1 (PMC3825294; doi:10.1007/s10482-013-0015-1)

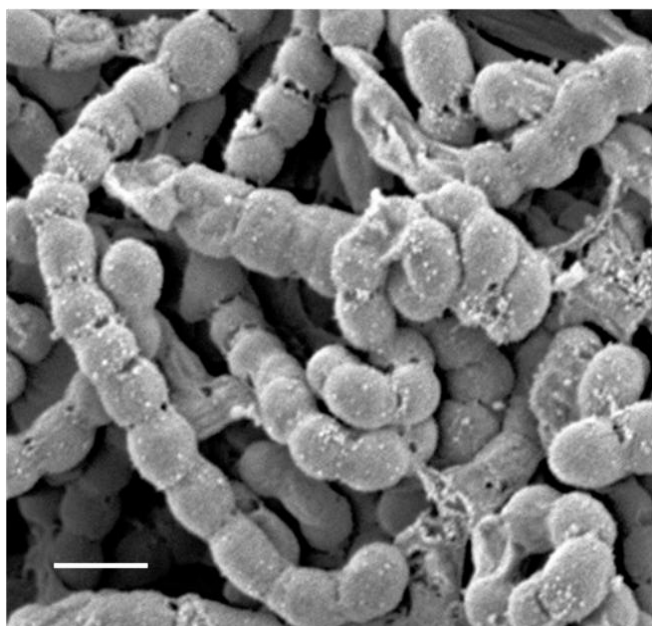

Supplement: Supplementary file 1 — Fig. 1. Two dimensional thin-layer chromatography of polar lipids of isolate HSCA14T stained with molybdenum blue (Sigma). Chloroform : methanol : water (32.5 : 12.5 : 2.0 v/v) were used in the first direction and chloroform : acetic acid : methanol : water (40 : 7.5 : 6 : 2 v/v) in the second direction. DPG, diphosphatidylglycerol; PE, phosphatidylethanolamine; PI, phosphatidylinositol; PIMS, phosphatidylinositol mannosides. Supplementary material 1 (PDF 133 kb) [file 10482_2013_15_MOESM1_ESM.pdf]

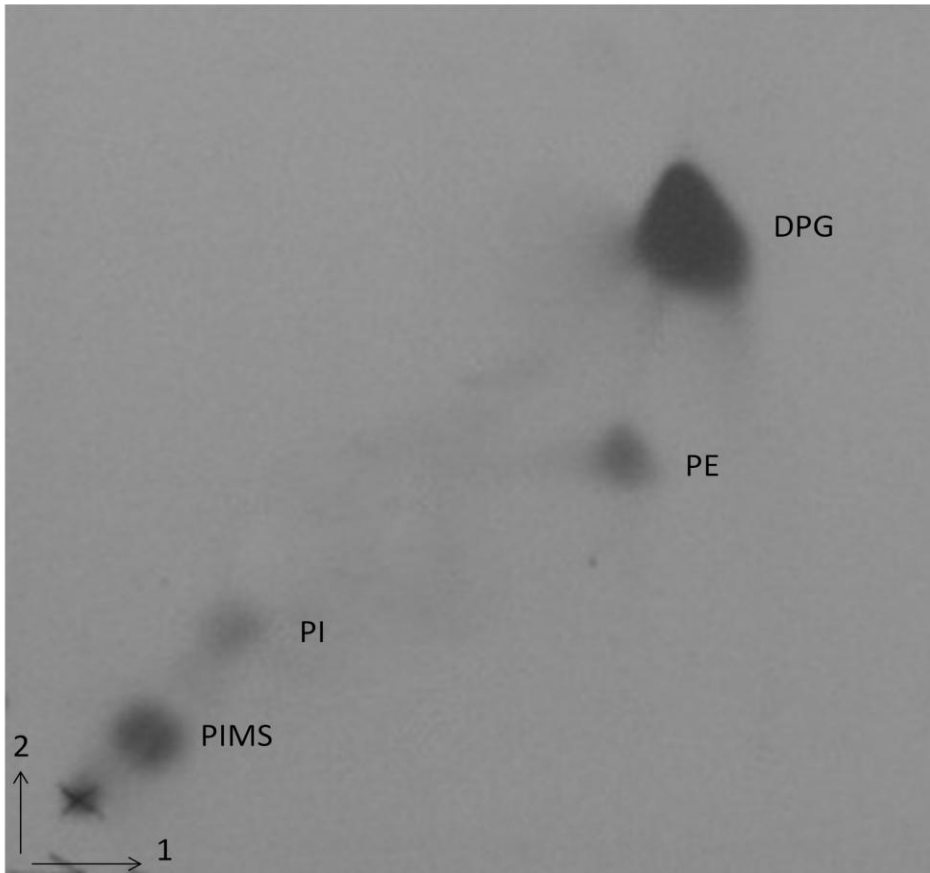

Supplement: Supplementary file 2 — Fig. 2. Scanning electron micrograph of isolate HSCA14T showing straight chains of smooth-surfaced, cylindrical spores on oatmeal agar after growth for 3 weeks at 28 °C. Bar, 1.0 μm. Supplementary material 2 (PDF 115 kb) [file 10482_2013_15_MOESM2_ESM.pdf]
